# Supplementary material for: Replacing the PDZ-interacting C-termini of DSCAM and DSCAML1 with epitope tags causes different phenotypic severity in different cell populations
Source: eLife. 2016 Sep 16;5:e16144. doi: 10.7554/eLife.16144 (PMC5026468; doi:10.7554/eLife.16144)
Supplement: Supplementary file 1. — p-values were calculated from each pairwise comparison across genotypes using the indicated measurement and test. NN = nearest neighbor analysis; PF = packing factor derived from DRP (density recovery profiling). DOI: http://dx.doi.org/10.7554/eLife.16144.018 [file elife-16144-supp1.docx]

| **Cell type** | **Measure** | **Test** | **Comparison** | **p value** |  | **Cell type** | **Measure** | **Test** | **Comparison** | **p value** |
| --- | --- | --- | --- | --- | --- | --- | --- | --- | --- | --- |
| bnos | Density | Tukey | ΔC to +/+ | 0.496 |  | Dab1 | Density | Tukey | ΔC to +/+ | 0.994 |
| bnos | Density | Tukey | ΔC to -/- | 0.023 |  | Dab1 | Density | Tukey | ΔC to -/- | 0.023 |
| bnos | Density | Tukey | -/- to +/+ | 0.004 |  | Dab1 | Density | Tukey | -/- to +/+ | 0.022 |
| bnos | Elo score | Wilcoxon | +/+ to Bax^-/-^ | 0.015 |  | Dab1 | NN | Tukey | ΔC to +/+ | 0.147 |
| bnos | Elo score | Wilcoxon | ΔC to Bax^-/-^ | 0.015 |  | Dab1 | NN | Tukey | ΔC to -/- | 0.004 |
| bnos | Elo score | Wilcoxon | ΔC to +/+ | 0.038 |  | Dab1 | NN | Tukey | -/- to +/+ | 0.000 |
| bnos | Elo score | Wilcoxon | ΔC to -/- | 0.017 |  | Dab1 | PF (DRP) | Tukey | ΔC to +/+ | 0.522 |
| bnos | Elo score | Wilcoxon | -/- to Bax^-/-^ | 0.017 |  | Dab1 | PF (DRP) | Tukey | ΔC to -/- | 0.414 |
| bnos | Elo score | Wilcoxon | -/- to +/+ | 0.017 |  | Dab1 | PF (DRP) | Tukey | -/- to +/+ | 0.063 |
| bnos | NN | Tukey | ΔC to +/+ | 0.780 |  | Dab1 | Voronoi | Tukey | ΔC to +/+ | 0.000 |
| bnos | NN | Tukey | ΔC to -/- | 0.050 |  | Dab1 | Voronoi | Tukey | ΔC to -/- | 0.000 |
| bnos | NN | Tukey | -/- to +/+ | 0.021 |  | Dab1 | Voronoi | Tukey | -/- to +/+ | 0.000 |
| bnos | PF (DRP) | Tukey | ΔC to +/+ | 0.584 |  | Cdh3-GFP | Density | Tukey | ΔC to +/+ | 0.195 |
| bnos | PF (DRP) | Tukey | ΔC to -/- | 0.141 |  | Cdh3-GFP | Density | Tukey | ΔC to -/- | 0.039 |
| bnos | PF (DRP) | Tukey | -/- to +/+ | 0.033 |  | Cdh3-GFP | Density | Tukey | -/- to +/+ | 0.001 |
| bnos | Voronoi | Tukey | ΔC to +/+ | 0.073 |  | Cdh3-GFP | Elo score | Wilcoxon | ΔC to +/+ | 0.108 |
| bnos | Voronoi | Tukey | ΔC to -/- | 0.158 |  | Cdh3-GFP | Elo score | Wilcoxon | ΔC to -/- | 0.016 |
| bnos | Voronoi | Tukey | -/- to +/+ | 0.004 |  | Cdh3-GFP | Elo score | Wilcoxon | -/- to +/+ | 0.007 |
| TH | Density | t-test | ΔC to Bax^-/-^ | 0.070 |  | Cdh3-GFP | NN | Tukey | ΔC to +/+ | 0.514 |
| TH | Density | Tukey | ΔC to +/+ | 0.000 |  | Cdh3-GFP | NN | Tukey | ΔC to -/- | 0.000 |
| TH | Density | Tukey | ΔC to -/- | 0.219 |  | Cdh3-GFP | NN | Tukey | -/- to +/+ | 0.000 |
| TH | Density | Tukey | -/- to +/+ | 0.004 |  | Cdh3-GFP | PF (DRP) | Tukey | ΔC to +/+ | 0.610 |
| TH | Elo score | Wilcoxon | +/+ to Bax^-/-^ | 0.235 |  | Cdh3-GFP | PF (DRP) | Tukey | ΔC to -/- | 0.573 |
| TH | Elo score | Wilcoxon | ΔC to Bax^-/-^ | 0.024 |  | Cdh3-GFP | PF (DRP) | Tukey | -/- to +/+ | 0.149 |
| TH | Elo score | Wilcoxon | ΔC to +/+ | 0.024 |  | Cdh3-GFP | Voronoi | Tukey | ΔC to +/+ | 0.671 |
| TH | Elo score | Wilcoxon | ΔC to -/- | 0.084 |  | Cdh3-GFP | Voronoi | Tukey | ΔC to -/- | 0.000 |
| TH | Elo score | Wilcoxon | -/- to Bax^-/-^ | 0.028 |  | Cdh3-GFP | Voronoi | Tukey | -/- to +/+ | 0.000 |
| TH | Elo score | Wilcoxon | -/- to +/+ | 0.030 |  | ipRGC | Density | t-test | ΔC to Bax^-/-^ | 0.076 |
| TH | NN | t-test | ΔC to Bax^-/-^ | 0.234 |  | ipRGC | Density | Tukey | ΔC to +/+ | 0.348 |
| TH | NN | Tukey | ΔC to +/+ | 0.000 |  | ipRGC | Density | Tukey | ΔC to -/- | 0.166 |
| TH | NN | Tukey | ΔC to -/- | 0.416 |  | ipRGC | Density | Tukey | -/- to +/+ | 0.007 |
| TH | NN | Tukey | -/- to +/+ | 0.001 |  | ipRGC | NN | t-test | ΔC to Bax^-/-^ | 0.341 |
| TH | PF (DRP) | t-test | ΔC to Bax^-/-^ | 0.372 |  | ipRGC | NN | Tukey | ΔC to +/+ | 0.004 |
| TH | PF (DRP) | Tukey | ΔC to +/+ | 0.001 |  | ipRGC | NN | Tukey | ΔC to -/- | 0.000 |
| TH | PF (DRP) | Tukey | ΔC to -/- | 0.580 |  | ipRGC | NN | Tukey | -/- to +/+ | 0.000 |
| TH | PF (DRP) | Tukey | -/- to +/+ | 0.006 |  | ipRGC | PF (DRP) | t-test | ΔC to Bax^-/-^ | 0.030 |
| TH | Voronoi | t-test | ΔC to Bax^-/-^ | 0.527 |  | ipRGC | PF (DRP) | Tukey | ΔC to +/+ | 0.000 |
| TH | Voronoi | Tukey | ΔC to +/+ | 0.128 |  | ipRGC | PF (DRP) | Tukey | ΔC to -/- | 0.976 |
| TH | Voronoi | Tukey | ΔC to -/- | 0.666 |  | ipRGC | PF (DRP) | Tukey | -/- to +/+ | 0.000 |
| TH | Voronoi | Tukey | -/- to +/+ | 0.036 |  | ipRGC | Voronoi | t-test | ΔC to Bax^-/-^ | 0.034 |
| VGLUT3 | Density | t-test | ΔC to Bax^-/-^ | 0.012 |  | ipRGC | Voronoi | Tukey | ΔC to +/+ | 0.057 |
| VGLUT3 | Density | Tukey | ΔC to +/+ | 0.000 |  | ipRGC | Voronoi | Tukey | ΔC to -/- | 0.000 |
| VGLUT3 | Density | Tukey | ΔC to -/- | 0.000 |  | ipRGC | Voronoi | Tukey | -/- to +/+ | 0.000 |
| VGLUT3 | Density | Tukey | -/- to +/+ | 0.000 |  | ipRGC-OFF | Elo score | Wilcoxon | +/+ to Bax^-/-^ | 0.005 |
| VGLUT3 | NN | t-test | ΔC to Bax^-/-^ | 0.000 |  | ipRGC-OFF | Elo score | Wilcoxon | ΔC to Bax^-/-^ | 0.860 |
| VGLUT3 | NN | Tukey | ΔC to +/+ | 0.029 |  | ipRGC-OFF | Elo score | Wilcoxon | ΔC to +/+ | 0.001 |
| VGLUT3 | NN | Tukey | ΔC to -/- | 0.009 |  | ipRGC-OFF | Elo score | Wilcoxon | ΔC to -/- | 0.004 |
| VGLUT3 | NN | Tukey | -/- to +/+ | 0.000 |  | ipRGC-OFF | Elo score | Wilcoxon | -/- to Bax^-/-^ | 0.098 |
| VGLUT3 | PF (DRP) | t-test | ΔC to Bax^-/-^ | 0.424 |  | ipRGC-OFF | Elo score | Wilcoxon | -/- to +/+ | 0.001 |
| VGLUT3 | PF (DRP) | Tukey | ΔC to +/+ | 0.000 |  | ipRGC-ON | Elo score | Wilcoxon | +/+ to Bax^-/-^ | 0.153 |
| VGLUT3 | PF (DRP) | Tukey | ΔC to -/- | 0.655 |  | ipRGC-ON | Elo score | Wilcoxon | ΔC to Bax^-/-^ | 0.680 |
| VGLUT3 | PF (DRP) | Tukey | -/- to +/+ | 0.002 |  | ipRGC-ON | Elo score | Wilcoxon | ΔC to +/+ | 0.125 |
| VGLUT3 | Voronoi | t-test | ΔC to Bax^-/-^ | 0.008 |  | ipRGC-ON | Elo score | Wilcoxon | ΔC to -/- | 0.018 |
| VGLUT3 | Voronoi | Tukey | ΔC to +/+ | 0.029 |  | ipRGC-ON | Elo score | Wilcoxon | -/- to Bax^-/-^ | 0.025 |
| VGLUT3 | Voronoi | Tukey | ΔC to -/- | 0.146 |  | ipRGC-ON | Elo score | Wilcoxon | -/- to +/+ | 0.002 |
| VGLUT3 | Voronoi | Tukey | -/- to +/+ | 0.000 |  |  | | | | |
